# The complete chloroplast genome of monotypic fern, *Mesopteris tonkinensis* (Thelypteridaceae)

**DOI:** 10.1080/23802359.2018.1502632

**Published:** 2018-08-17

**Authors:** Zhe Ding, Tianjian Chen, Shanshan Liu, Shufeng Li, Zhen Wang, Ting Wang, Yingjuan Su

**Affiliations:** a School of Life Sciences, Sun Yat-sen University, Guangzhou, China;; b College of Life Sciences, Nanjing Agricultural University, Nanjing, China;; c College of Life Sciences, South China Agricultural University, Guangzhou, China;; d Research Institute of Sun Yat-sen University in Shenzhen, Shenzhen, China

**Keywords:** *Mesopteris tonkinensis*, monotypic fern, chloroplast genome, phylogenetic analysis

## Abstract

*Mesopteris tonkinensis* is monotypic species in the genus *Mesopteris* (Thelypteridaceae). We characterized its complete chloroplast genome sequences by Illumina sequencing and *de novo* assembly. The genome size is 161,380 bp in length with a GC content of 43.6%, containing a large single-copy (LSC) region of 82,678 bp, a small single-copy (SSC) region of 21,786 bp and a pair of inverted repeat (IR) of 28,458 bp. In total, 131 genes are identified, including 88 protein-coding genes, 34 tRNA genes with absent *trnV-UAC*, eight rRNA genes and one pseudogene. ML tree revealed that *M. tonkinensis* and *Christella appendiculata* were closely related.

*Mesopteris tonkinensis* is monotypic species in the genus *Mesopteris* (Thelypteridaceae) (Lin et al. 2013). It is endemic to wet rocks in limestone areas in South China and North Vietnam (Ching 1978). The feature of this fern reflects in callose protuberance at the sinuses and 2½ pairs of veinlets connivent under the sinuses (He and Zhang 2012). Established since 1934, *M. tonkinensis* was successively ascribed to *Dryopteris* (Dryopteridaceae), *Thelypteris*, *Lastrea* and *Amphineuron* (Thelypteridaceae) due to its unique characteristics (Wang et al. 2015). The first molecular phylogenetic analysis strongly supported *M. tonkinensis* as monophyletic and as a part of the *Cyclosorus* clade (He and Zhang 2012). Because of its inaccessibility, the research of *M. tonkinensis* is only limited in morphology, cytology and phylogeny (Huang and Zhou 1994; He and Zhang 2012; Wang et al. 2015). Hence, it is necessary to acquire whole chloroplast (cp) genome sequence of *M. tonkinensis*, which provide more useful information to determine its classification position.

A plant material of *M. tonkinensis* was provided by South China Botanical Garden, Chinese Academy of Sciences (23°11′3.56″N, 113°21′43.28″E) and saved in Herbarium of Sun Yat-sen University (SYS; voucher: *SS Liu 201617*). Genomic DNA was extracted from fresh leaves by Tiangen Plant Genomic DNA Kit (Tiangen Biotech Co., Beijing, China) and broken into 300 bp with Covaris M220 (Covaris Inc., MS, USA). A paired-end (PE) library was constructed using NEBNext Ultra DNA Library Prep Kit for Illumina (New England BioLabs Inc., Ipswich, MA). After sequencing was performed in Illumina Hiseq 2500 platform (Illumina Inc., San Diego, USA), we removed the adapters and low-quality reads from 2.38G raw data through Trimmomatic v0.32 (Bolger et al. 2014) and qualified 2.05G clean data by FastQC v0.10.0 (Andrews 2010). The complete chloroplast genome was *de novo* assembled by Velvet v1.2.07 (Zerbino and Birney 2008), and further annotated using DOGMA (Wyman et al. 2004) and tRNAscan-SE (Schattner et al. 2005), and finally manual confirmation based on BLAST searches. In order to further analyze phylogenetic relationships, ten ferns including *Ophioglossum californicum* as outgroup were selected and aligned with MAFFT v.7.221 (Katoh and Standley 2013). Phylogenetic tree was generated by a maximum likelihood analysis of RAxML v8.0 (Stamatakis 2014) with 1000 bootstrap replicates.

The complete chloroplast genome of *M. tonkinensis* is a typical quadripartite structure with 161,380 bp, which was separated into a large single-copy (LSC) region of 82,678 bp, a small single-copy (SSC) region of 21,786 bp and a pair of inverted repeat (IR) regions of 28,458 bp (GenBank accession number: MH500229). Whole genome presents the GC content of 43.6%, whereas corresponding GC value in LSC, SSC, and IR is 42.2%, 39.87%, and 46.9%, respectively. The genome contains 88 protein-coding genes, 34 tRNA genes with absent *trnV-UAC*, eight rRNA genes and one pseudogene (*ndhB*). Among these genes, 116 genes are single-copy genes, 15 genes (*ndhB*, *rps16*, *atpF*, *rpoC1*, *petB*, *petD*, *ndhA*, *rpl16*, *rpl2*, *trnG-UCC*, *trnV-UAC*, *trnA-UGC*, *trnI-GAU*, *trnL-UAA*, and *trnT-UGU*) harbor a single intron, and three genes (*ycf3*, *clpP*, and *rps12*) have two introns. ML tree revealed that *M. tonkinensis* and *Christella appendiculata* were closely related with high bootstrap support values (Figure 1).

**Figure 1.**
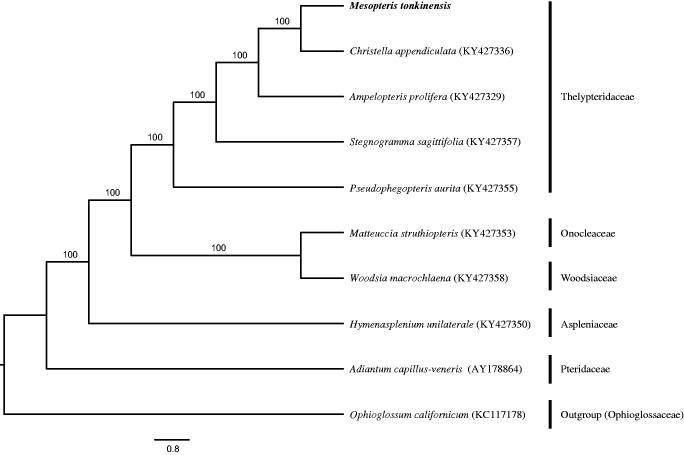
Phylogenetic tree of ten ferns generated from whole chloroplast genome sequences using RAxML v8.0 with maximum likelihood analysis. *Ophioglossum californicum* was set as outgroup. Bootstrap support values from 1000 replicates are indicated in the nodes of phylogenetic tree.
